# Do you see what I see? Mobile eye-tracker contextual analysis and inter-rater reliability

**DOI:** 10.1007/s11517-017-1669-z

**Published:** 2017-07-15

**Authors:** S. Stuart, D. Hunt, J. Nell, A. Godfrey, J. M. Hausdorff, L. Rochester, L. Alcock

**Affiliations:** 1 Institute of Neuroscience/Newcastle University Institute for Ageing, Clinical Ageing Research Unit, Newcastle University, Newcastle upon Tyne, NE4 5PL UK; 2 Newcastle upon Tyne Hospitals NHS foundation trust, Newcastle upon Tyne, UK; 3 Center for Study of Movement, Cognition and Mobility, Neurological Institute, Tel Aviv Sourasky Medical Center, Tel Aviv, Israel; 4 Rush Alzheimer’s Disease Center and Department of Orthopaedic Surgery, Rush University Medical Center, Chicago, IL USA; 5 Sagol School of Neuroscience and Department of Physical Therapy, Sackler Faculty of Medicine, Tel Aviv University, Tel Aviv, Israel

**Keywords:** Eye-tracking, Contextual, Older adults, Parkinson’s disease, Algorithm, Inter-rater

## Abstract

Mobile eye-trackers are currently used during real-world tasks (e.g. gait) to monitor visual and cognitive processes, particularly in ageing and Parkinson’s disease (PD). However, contextual analysis involving fixation locations during such tasks is rarely performed due to its complexity. This study adapted a validated algorithm and developed a classification method to semi-automate contextual analysis of mobile eye-tracking data. We further assessed inter-rater reliability of the proposed classification method. A mobile eye-tracker recorded eye-movements during walking in five healthy older adult controls (HC) and five people with PD. Fixations were identified using a previously validated algorithm, which was adapted to provide still images of fixation locations (*n* = 116). The fixation location was manually identified by two raters (DH, JN), who classified the locations. Cohen’s kappa correlation coefficients determined the inter-rater reliability. The algorithm successfully provided still images for each fixation, allowing manual contextual analysis to be performed. The inter-rater reliability for classifying the fixation location was high for both PD (kappa = 0.80, 95% agreement) and HC groups (kappa = 0.80, 91% agreement), which indicated a reliable classification method. This study developed a reliable semi-automated contextual analysis method for gait studies in HC and PD. Future studies could adapt this methodology for various gait-related eye-tracking studies.

## Introduction

Eye-tracking during real-world tasks is increasingly popular within various fields of research, including neurology [1], psychiatry [2] and human movement science [3]. Eye-movements can be broken into two classifications: saccadic fast eye-movements which shift foveation between different areas of interest within the environment, and fixation eye-movements (including smooth pursuits) where the eye pauses on areas of interest [4]. Increased popularity in recording eye-movements (particularly saccades) is due to their known relationships with cognitive and visual processes [5], allowing inferences regarding impairment of these underlying functions. Describing eye-movements during real-world tasks (i.e. walking, driving, obstacle crossing) is important to understand visuo-cognitive impairment and develop effective interventions in ageing and neurodegenerative disorders such as Parkinson’s disease (PD).

Eye-tracking technology has progressed from static devices with high resolutions (>200 Hz), to mobile systems which sacrifice resolution (50–60 Hz) in exchange for mobility [3]. Mobile infrared or video-based eye-trackers provide comprehensive recording of temporal and spatial features of eye-movements during real-world tasks. Mobile eye-tracking devices have been used in older adult and PD research [6]; however, a recent review highlighted a number of limitations [3]. For example, currently, little focus has been placed on contextual outcomes (i.e. what participants are looking at or areas of interest) during real-word tasks in older adults and people with PD, which may provide clinically relevant information such as whether individuals look at task-relevant or hazardous areas.

Eye-tracker manufacturers have attempted to automate contextual analysis within their software (such as iMotions Inc., Boston, MA and D-Lab, Ergoneers GmbH, Germany) using heat maps (i.e. displaying contextual data by a colour scale) or pre-defined object targeting using environmental markers or pixel-based analysis [7–11]. Current systems are used during static testing (e.g. reading [12] or image viewing [13, 14] or video viewing [15]) and require manual input of information about the visual scene [13, 16] or about specific objects within the scene (e.g. facial detection [17, 18] or shopping products [19]). The restricted nature of such automatic analysis means that they have limited application for assessment of dynamic real-world activities (e.g. walking). Further, such techniques have not been validated and present methodological issues. For example, environmental markers may distract gaze and impact results. Similarly, heat maps may be impacted by eye-tracker accuracy or resolution [5] and often require an initial still frame to overlay the heat map onto, which may not represent a full mobile trial when walking. Thus, accuracy of current automated contextual analysis is questionable, particularly during real-world tasks.

Existing contextual analysis has been limited to manual frame-by-frame video processing, which has been conducted in healthy adults [20–22] and PD [23–25] during various activities (i.e. walking, visual cues, within a flight simulator). Such studies have reported limited contextual data, such as whether individuals are looking at the floor, a doorway or side walls [24]. Limited information on analysis has also been provided (e.g. fixation classification), but no previous study has assessed their manual contextual analysis method. Manual analysis can be entirely subjective, time consuming and not feasible for studies involving large cohorts (i.e. studies often perform analysis on a sub-group). Contextual analysis has potential to provide an increased level of detail regarding task performance, therefore development and examination of contextual methodologies is paramount.

This study aimed to assess inter-rater reliability of semi-automated mobile eye-tracker contextual analysis of data obtained during various walking tasks in older adults and people with PD. A validated mobile eye-tracker algorithm [26] provided fixation data (e.g. timing) and was adapted to extract still images of fixation locations. A classification method was provided to two raters to define fixation locations within the visual scene, which was then evaluated.

## Methods

### Participants

Eye-tracking data from five healthy control older adults (HC) and five people with PD were randomly selected from two larger studies at the Clinical Ageing Research Unit, Newcastle University, which were approved by the local NHS ethics committee (research ethics committee (REC) ref 13/NE/0128, REC ref 12/NE/0249). The first study was ‘Vision and gait in Parkinson’s disease: impact of cognition and response to visual cues’ that investigated eye-movements during walking (with doorways, turns, visual cues, dual tasks etc.) in older adults and people with Parkinson’s disease. The second study was a site-specific sub-study from ‘V-TIME: A treadmill training program augmented by virtual reality to decrease fall risk in older adults’, which investigated eye-movements during obstacle crossing in Parkinson’s disease and older adult fallers. Written informed consent was obtained from each participant.

### Protocol

A Dikablis mobile eye-tracker (Ergoneers GmbH, Germany) was used to record eye-movements during gait at a sampling rate of 50 Hz, with an accuracy of ~1.2° [27]. The Dikablis utilised two cameras: a monocular infrared camera that recorded participant gaze co-ordinates and a central, forward facing fish-eye camera captured the participant visual field. The manufacturer’s four-point calibration procedure was used to calibrate the view of the two cameras, which were overlaid and showed a crosshair representing pupil location within the visual field of view.

The participants were asked to walk in a straight line through an uncluttered gait laboratory over a distance of 7 m under three task conditions: straight walk, straight walk with a visual cue and straight walk with an obstacle (over a GAITRite mat) [28]. The visual cue consisted of five black taped cues beginning 150 cm from the starting location and spaced by 50 cm, and the obstacle was a yellow 15 × 2 × 60 cm board. Both were of high contrast to the floor. The individuals were instructed to step over the visual cues or obstacle when completing the walks and each participant performed three walks per condition.

### Data processing and analysis

#### Data processing and algorithm analysis

The first trial from the three different walking conditions (straight, visual cue and obstacle) was processed for each participant (five older adults and five PD per condition; 30 videos in total).

**Fig. 1:**
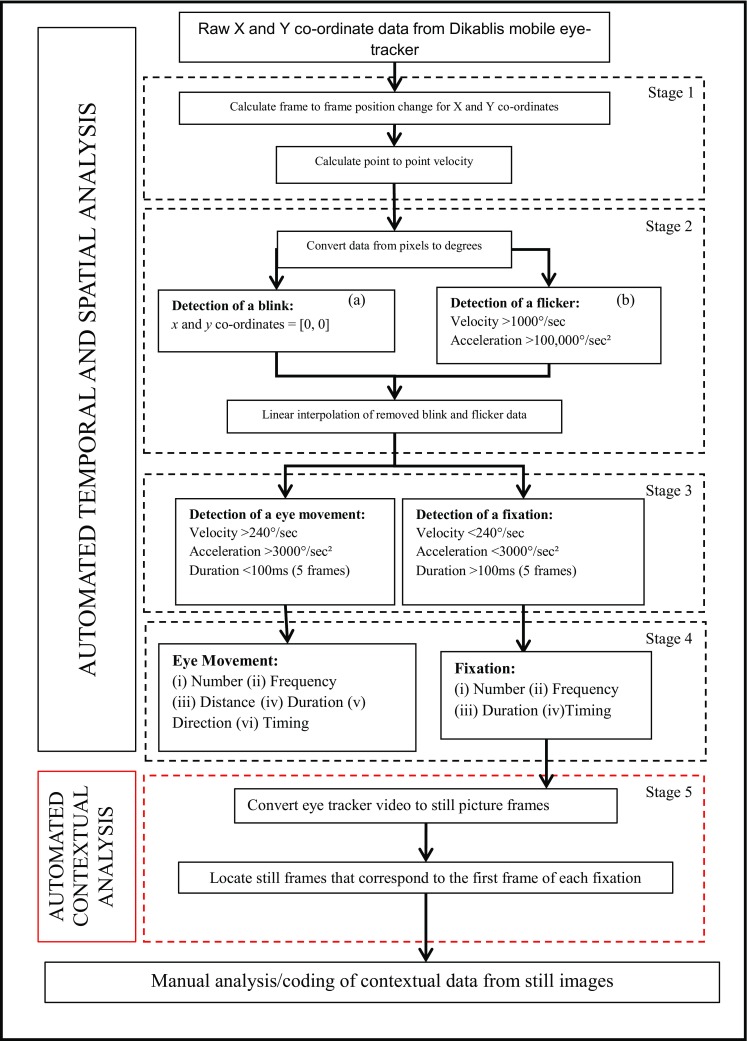
Algorithm and manual analysis flow chart: adapted from [26], contextual analysis procedures are represented in *red boxes*

**Fig. 2:**
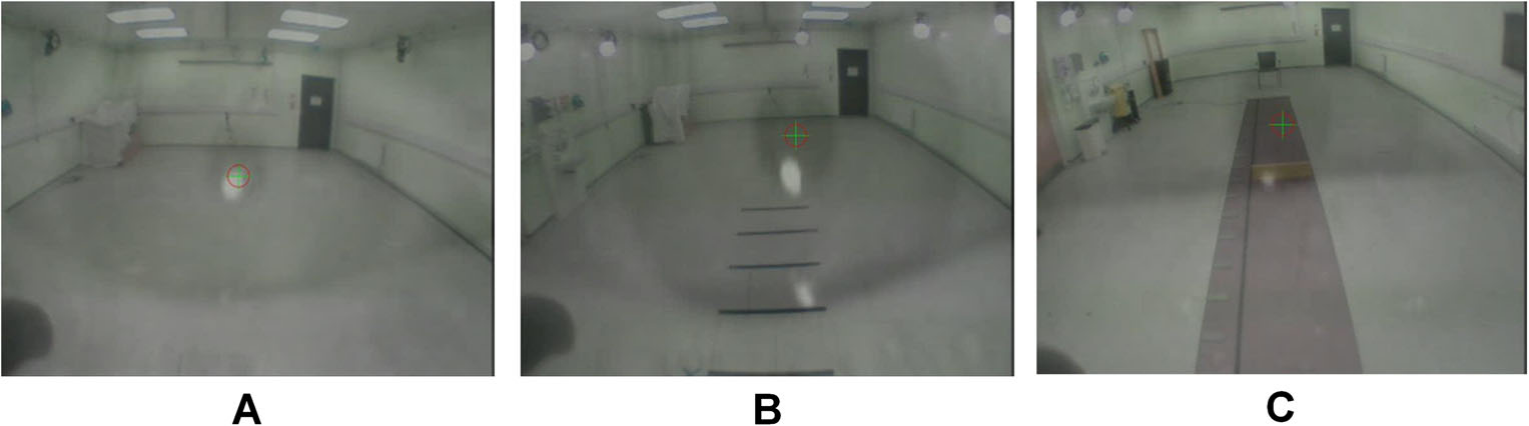
Examples of fixations made during **a** straight walking, **b** visual cueing and **c** obstacle crossing tasks

First, raw data was processed using the manufacturer’s software (Dikablis Analysis 2.5, Ergoneers GmbH, Germany). This involved manual interpolation (frame-by-frame) of the eye-tracker video footage to locate the centre of the pupil in any frame which the software had failed to locate it. Inaccurate pupil location detections were manually corrected. Second, video footage was manually cropped to the length of the walking trial and exported for temporal and spatial analysis, and final contextual analysis. Third, a previously validated mobile eye-tracker algorithm [26] was used to perform the temporal and spatial analysis of the eye-tracker data (stages 1–4, Fig. 1), providing eye-movement and fixation outcomes (stage 4, Fig. 1). Importantly this automated step provided the starting frame of each eye-movement and fixation. Finally, contextual analysis involved initially converting eye-tracker overlaid eye and scene camera video collected during the walking tasks into individual photographic images for each frame of the video. This was done using the VideoReader function in MATLAB^®^. Once the video data had been analysed, still images (.jpg format) of the start of each fixation were exported (depicted in Fig. 2).

#### Manual fixation location analysis and inter-rater reliability

The fixation frames for each participant were analysed by two separate raters (DH, JN), who used a pre-defined classification method to categorise participant fixation location for the 116 total fixations identified (Table 1). Each rater viewed the images of fixations for each participant (*n* = 10) provided by the algorithm and used the classification method to code the area within which they judged that each fixation had been made. The number of fixations made in the various locations by the participants during each of the tasks was then compared between that of the raters.

**Table 1:** Classification of fixation locations

Fixation location	Code	Definition
Wall straight	1	The wall in front of the participant within the width of the task area
Side wall	2	The walls to either side of the task area
Near floor ahead	3	The floor within 2 m of the participant, approximated to 3 paces
Far floor ahead	4	The floor beyond 2 m of the participant, approximated to 3 paces
Side floor	5	The floor area to either side of the task area
Ceiling	6	The ceiling
Obstacle	7	The obstacle^a^
Near cue	8	The cued area within 2 m from the participant, approximated to 3 paces^a^
Far cue	9	The cued area beyond 2 m from the participant, approximated to 3 paces^a^

^a^Condition-specific areas of interest that did not apply to the unobstructed gait trials

**Fig. 3:**
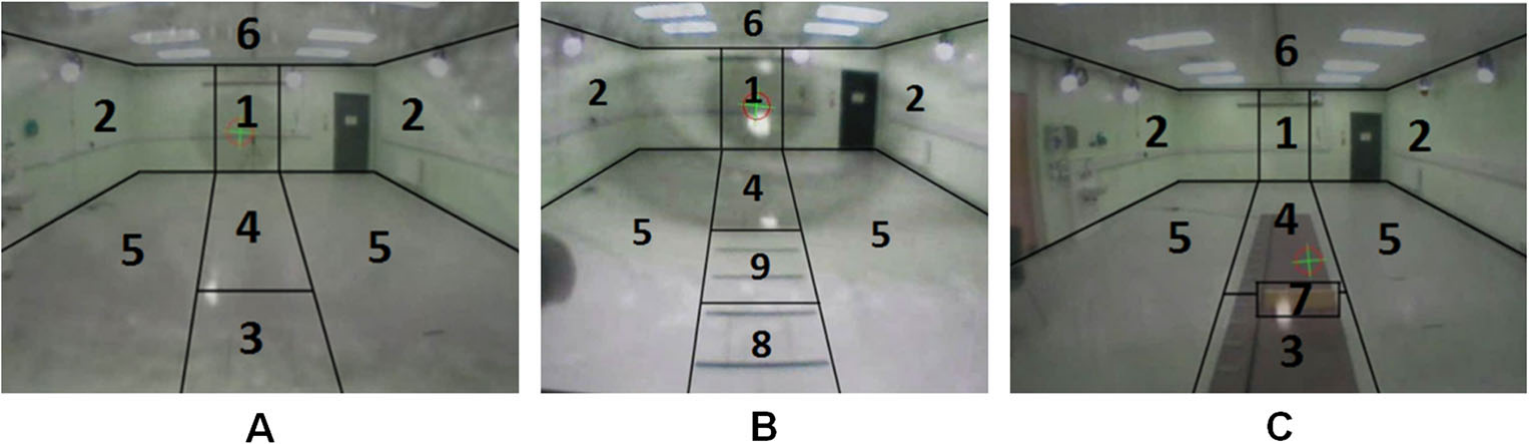
Example frame of fixation location classification in **a** straight walking, **b** visual cueing and **c** obstacle tasks. Fixation locations as defined by Table 1 are identified by their *coded number*

The definitions of the areas of fixation location are presented in Table 1. Within the classification method, ‘task area’ was defined as the pathway between the participant and the wall at the end of the laboratory with a width approximated to that of the cues and obstacle (Fig. 3). The ‘cued area’ was defined as the black taped cues, the area of the floor between each of them and a 50-cm area beyond the final cue. Figure 3 displays an example frame for each task condition and the boundaries which demarcate the locations presented in Table 1.

### Statistical analysis

Statistical analysis was performed using SPSS v.21.0 (IBM Corp., Armonk, NY). Data were assessed for normality using Kolmogorov-Smirnov tests. Between-group comparisons were not performed as identifying pathology-associated differences was not the focus of this study. Inter-rater agreement was analysed using descriptive data (i.e. agreement between the raters on the number and percentage of fixation locations) and Cohen’s kappa coefficient (Cohen, 1960). Kappa agreement was interpreted as follows: poor <0.20, fair 0.20 to 0.40, moderate, 0.40 to 0.60, good 0.60 to 0.80 and excellent 0.80 to 1.00 (Field, 2013).

## Results

The adapted mobile eye-tracker algorithm produced 116 still images of fixation locations from data obtained when walking in HC and PD participants. Inter-rater reliability results for fixation location identification are displayed in Tables 2 and 3. The inter-rater reliability of identifying fixation location had excellent comparability between the two groups (PD *n* = 5, kappa = 0.80, HC *n* = 5, kappa = 0.80). On average, the raters agreed upon 95% of fixation locations in the PD group and 91% in HC group. The total number of fixation locations not agreed upon was relatively low, 9 frames of 116 inspected. The differences were primarily seen within visual cue and floor fixations (*n* = 9 in total).

**Table 2:** Inter-rater reliability for PD participants

	Participant									
	PD 1		PD 2		PD 3		PD 4		PD 5	
Fixation location	R1	R2	R1	R2	R1	R2	R1	R2	R1	R2
Wall (straight ahead): *n*	5	5	4	4	5	5	3	3	4	4
Wall (side): *n*	2	2	0	0	0	0	0	0	1	1
Floor (near, straight ahead): *n*	2	2	0	0	1	1	0	0	1	1
Floor (far, straight ahead): *n*	3	3	1	1	1	1	1	1	4	4
Floor (side): *n*	4	3	0	0	0	0	0	0	0	0
Obstacle: *n*	0	0	0	0	2	2	0	0	0	0
Visual cue (near): *n*	1	2	0	0	2	1	2	2	4	5
Visual cue (far): *n*	0	0	0	0	0	1	1	1	1	0
Agreed locations: *n* (%)	16 (94%)		5 (100%)		10 (91%)		7 (100%)		14 (93%)	
Not agreed location: *n* (%)	1 (6%)		0 (0%)		1 (9%)		0 (0%)		1 (7%)	

*PD* Parkinson’s disease, *R1* rater one, *R2* rater two

**Table 3:** Inter-rater reliability for HC participants

	Participant									
	HC 1		HC 2		HC 3		HC 4		HC 5	
Fixation location	R1	R2	R1	R2	R1	R2	R1	R2	R1	R2
Wall (straight ahead): *n*	9	9	5	5	8	8	4	4	4	4
Wall (side): *n*	2	2	0	0	0	0	0	0	0	0
Floor (near, straight ahead): *n*	1	4	1	0	1	1	0	0	3	3
Floor (far, straight ahead): *n*	6	3	0	0	4	4	1	1	5	5
Floor (side): *n*	0	0	0	0	0	0	0	0	0	0
Obstacle: *n*	1	1	0	0	0	0	1	1	0	0
Visual cue (near): *n*	0	0	3	4	2	2	1	1	1	3
Visual cue (far): *n*	0	0	0	0	0	0	1	1	2	0
Agreed locations: *n* (%)	16 (84%)		8 (89%)		15 (100%)		8 (100%)		13 (87%)	
Not agreed location: *n* (%)	3 (16%)		1 (11%)		0 (0%)		0 (0%)		2 (13%)	

*HC* healthy control, *R1* rater one, *R2* rater two

## Discussion

This study aimed to develop and implement a methodology to semi-automate the contextual analysis of mobile eye-tracking data collected during gait in HC and PD participants, and examine the reliability of this process. We adapted our previous mobile eye-tracker algorithm [26] to provide still fixation images for further analysis, and developed a classification method to objectively quantify the contextual information of fixation locations (areas of interest). This study provides a simple, reliable methodology applicable to mobile eye-tracker data obtained during real-world tasks, such as walking.

### Development and inter-reliability of a classification method

Our previous algorithm [26] was successfully adapted to provide still images of fixation locations which could then be used for manual contextual analysis. The addition of this step (step 5, Fig. 1) to the automated algorithm saves time in the processing of fixation location data and reduces some of the subjectivity in the process, as a quantitative algorithm is used to locate the start of fixations. The agreement between the two independent raters alleviates any concerns regarding rater bias. This development would allow for large datasets to be processed and analysed in shorter periods of time than has been possible prior to this study, therefore more in-depth analysis of fixation locations during walking in older adults and people with PD (and other populations) may be performed.

A pre-defined fixation location classification method (Table 1) provided standardised criteria to identify fixations when walking under various conditions. The classification method split the visual field into nine areas of interest (Fig. 3), providing greater detail (i.e. more areas) than previous studies [24] (e.g. door, floor and ahead). Eye-tracking was examined during a dynamic task, therefore a large volume was used for each area to account for eye-tracker limitations and apparent changes in object (obstacle or visual cue) sizes when viewed from the scene camera during walking (i.e. the larger, the closer a participant gets to the object). Separating the contextual data into smaller areas (such as individual visual cue lines) would likely have introduced more variability in fixation location [5]. Although previous research has reported more specific outcomes (such as participants looking two steps ahead) [25], limited information on accuracy and reliability of eye-tracking devices raises questions regarding interpretation of contextual data. For example, an eye-tracker with poor accuracy may incorrectly show that the pupil location crosshair is in an area that the individual is not looking at.

In the present study, the participants completed the same walking tasks in a laboratory environment and data were analysed using the same algorithm and classification method, and fixation location comparisons were recorded by two independent raters. Under these conditions, the classification method was found to be highly reliable, determined by the kappa correlation coefficient, which was 0.80 in PD and 0.80 in HC participants. Although reliability was excellent, there were a small number of inter-rater fixation location disagreements (*n* = 9). With the exception of one pair of differing results (near cue/side floor), the disputed fixation locations were from areas close to the margins of the defined classified areas (i.e. near or far, within or outside of the cueing area boundary), and as such were susceptible to subjective interpretation. However, given the reliability shown across the three walking conditions, we suggest that this classification method would be suitable for use with these tasks or other similar tasks where the classification method could be adapted and employed. Our algorithm used the first still frame of a fixation location and large classification areas to account for long fixations while walking that may move through areas. Future studies that wish to examine smaller areas or locations may require further still images from the fixation data to classify locations.

## Conclusions

We successfully adapted a validated mobile eye-tracker algorithm and created a simple but reliable classification method to semi-automate contextual data analysis (i.e. fixation locations) of data obtained during various walking tasks in HC and PD. Our methodology may be useful for other studies interested in analysing contextual information from mobile eye-tracking data obtained during walking.
